# Detecting Happiness Using Hyperspectral Imaging Technology

**DOI:** 10.1155/2019/1965789

**Published:** 2019-01-15

**Authors:** Min Hao, Guangyuan Liu, Anu Gokhale, Ya Xu, Rui Chen

**Affiliations:** ^1^School of Electronic and Information Engineering, Southwest University, Chongqing, China; ^2^Chongqing Key Laboratory of Non-linear Circuit and Intelligent Information Processing, Southwest University, Chongqing, China; ^3^Illinois State University, Normal, IL, USA; ^4^Center of Technical Support for Network Security, Chongqing Municipal Public Security Bureau, Chongqing, China; ^5^College of Computer and Information Science, Southwest University, Chongqing, China

## Abstract

Hyperspectral imaging (HSI) technology can be used to detect human emotions based on the power of material discrimination from their faces. In this paper, HSI is used to remotely sense and distinguish blood chromophores in facial tissues and acquire an evaluation indicator (tissue oxygen saturation, StO_2_) using an optical absorption model. This study explored facial analysis while people were showing spontaneous expressions of happiness during social interaction. Happiness, as a psychological emotion, has been shown to be strongly linked to other activities such as physiological reaction and facial expression. Moreover, facial expression as a communicative motor behavior likely arises from musculoskeletal anatomy, neuromuscular activity, and individual personality. This paper quantified the neuromotor movements of tissues surrounding some regions of interest (ROIs) on smiling happily. Next, we selected six regions—the forehead, eye, nose, cheek, mouth, and chin—according to a facial action coding system (FACS). Nineteen segments were subsequently partitioned from the above ROIs. The affective data (StO_2_) of 23 young adults were acquired by HSI while the participants expressed emotions (calm or happy), and these were used to compare the significant differences in the variations of StO_2_ between the different ROIs through repeated measures analysis of variance. Results demonstrate that happiness causes different distributions in the variations of StO_2_ for the above ROIs; these are explained in depth in the article. This study establishes that facial tissue oxygen saturation is a valid and reliable physiological indicator of happiness and merits further research.

## 1. Introduction

There is a growing interest in the more positive emotions such as happiness [1–3]. Moreover, a state of happiness can overcome negative emotions such as stress [4, 5]. When people are engaged in social experiences that make them feel happy, people may exhibit measurable physiological characteristics such as blushing, facial expression features such as smiling, and body behavior such as dancing. With the exception of facial expression, specifically, happiness presumably includes a greater involvement of, for example, human physiology, psychology, behavior, and other human factors. Comprising a complex mix of behavioral, facial, physiological, and psychological traits, happiness is suspected to play a key role in many fields, including task execution [6, 7], healthcare [8, 9], and teaching and learning [10, 11].

## 2. Review of the Literature

Various in-depth studies have explored human emotions in terms of facial expressions. Witt and Flores-Mir [12, 13] and Janson et al. [14] investigated the facial smile paradigm by observing the subjects' lips and dentition. Arigbabu et al. [15] investigated smile detection from face images in unconstrained environments. A proposed framework provided a very competitive detection rate and exploited image alignment as an important stage for improving the performance of smile detection. Of course, the studies based on facial expressions mostly are based on the hypothesis that smile is often assumed to indicate happiness, which contradicts the fact that smile sometimes is pretended to deliberately adapt to a certain subjective understanding, which is actually not happiness.

Physiological signals is another significant entity that is studied in the field of emotional research. It is noteworthy that physiological signals are not influenced by human subjective consciousness. In addition, emotional reactions have been found to cause fluctuations in a variety of physiological indicators used to measure nervous activities. Kreibig [16] summarized 134 studies and found significant differentiation between the following physiological indices: heart rate (HR, the most commonly used indicator), skin conductance (SC), blood pressure (BP), and respiration rate (RR). Using images or videotapes as emotional stimuli, Codispoti et al. [17] and Bianchin and Angrilli [18] found that the heart rate (HR) was slower in positive emotional states than in neutral states. Gendolla et al. [19] and Neumann and Waldstein [20] reported that significantly higher systolic blood pressure (SBP) occurred in negative emotional states rather than in positive states. Using the multivariant correlation method, Wen et al. [21] analyzed the affective physiological changes in multisubject galvanic skin response (GSR), the first derivative of GSR (FD_GSR) and HR, and obtained an overall accuracy rate of 74% for quinary classification of amusement, anger, grief, fear, and the baseline state. Von Leupoldt and Dahme [22] verified that both positive and negative emotions can lead to respiratory resistance because of airway constriction when a picture stimulus pattern is introduced. Vianna and Tranel [23] found a higher positive correlation (*r* = 0.64) between the peak amplitude of the electrogastrogram (EGG) and the degree of arousal assessed subjectively by film-elicited methods. Pavlidis et al. [24] quantified the perspiration responses in the perinasal area—regarded as a physiological phenomenon. In addition, these authors introduced thermal imaging (TI) technology for the unobtrusive detection of perspiration signals.

Traditionally, various classic physiological indicators, including HR, BP, GSR, respiration, and EGG, require diverse contact sensors to measure changes in body responses. The inconvenience of applying these measuring techniques to certain fields in particular motivated researchers to investigate more reliable assessment methods using noninvasive technology. Thus, more attention has been paid to contactless detection technologies such as TI and hyperspectral imaging (HSI). These instruments are unobtrusive and require minimal interaction between subjects and examiners. The two imaging technologies, TI and HSI, allow measurement of physiological features such as blood flow, pulse rate, and breathing rate by spectral technologies. TI is directly related to tissue blood flow and eventually detects the changes in skin temperature and perspiration responses of the targeted regions. However, the technology becomes considerably less reliable when the ambient temperature changes suddenly. Consequently, we selected an alternative technique, HSI, to measure the affective features.

## 3. HSI Technique

HSI enables the imaging of a scene in hundreds of contiguous, narrow wavebands, with a bandwidth of approximately 10 nm, particularly in the visible and infrared regions of the electromagnetic spectrum, to form image cubes with both spatial and spectral dimensions [25, 26]. Due to the nature of light reflection by the object in the scene, HSI can capture the intensity of the reflected light within a narrow slice of wavebands across the whole spectrum and transform the extent in terms of pixels. Compared with conventional photography, HSI uses a narrow bandwidth for spectral sensing, enhancing the color discrimination ability. Its power of material discrimination is the reason why HSI is used as the primary technique in this research.

In this paper, HSI instrument employed records the imaging only for one row line of an object at a time—this only requires the scanning movement of HSI camera. Each of the recording process consists of the imaging under different wavebands within the whole spectrum. Next, it constructs a two-dimensional image (spatial and spectral axis). With a predetermined scanning frequency, it can obtain the total image slices of the object. Then, a 3D cube can be created by stacking all the 2D slices in sequence. Therefore, depending on the configuration, HSI can be introduced to remotely sense and discriminate blood chromophores from body tissues; the amount of oxygenation within the blood is subsequently quantified using an optical absorption model.

To recap, the HSI method is a noncontact detection technology for detecting physiological signals. Because of the power of material discrimination, HSI technology has increasingly been explored in various areas of research [27–29]. As a technology of longer spectrum width and higher imaging precision, HSI was utilized directly to remotely sense StO_2_ [30–32]. Thus, HSI is convenient to both subjects and testers. Additionally, this method can effectively counteract the aforementioned defects remaining in TI, and most important of all, it is not easily affected by environmental uncertainties.

## 4. Purpose of the Study

This study is based on the hypothesis that a happy state of mind results in measurable physiological changes that can ascertain whether someone is experiencing genuine happiness. The authors introduce facial tissue oxygen saturation (StO_2_), an innovative physiological measure to evaluate the effects of happiness on facial tissues by using HSI technology and test the reliability and repeatability of these measures.

The aims of this study were to examine (1) whether happiness causes fluctuations of affective signal (StO_2_) in facial regions, (2) the affective meanings of the changes in the neurophysiological movements of tissues using StO_2_ as a measure, and (3) the relative distribution pattern of StO_2_ for six facial regions of interest (ROIs)**—**forehead, eye, nose, cheek, mouth, and chin**—**when smiling happily.

## 5. Methodology

This section describes the population and sample for the study, research design, and data analysis. The study used two distinct self-reporting questionnaires and an interview with the expert to arrive at the sample from the population for the study. The process is explained in the following sections.

### 5.1. Population and Sample

Twenty-six healthy young-adult volunteers (16 females) participated in this study. The sample size for determining the main and interactive effects of emotion and ROI was computed to achieve a priority power = 0.80 and detection of medium effect sizes Cohen's f, calculated by GPower. None of the participants had known functional insufficiencies, and all participants were selected from Southwest University of Chongqing, China. After receiving a full description of the study, all volunteers gave written informed consent. They were paid a fixed amount (¥30) to participate. The inclusion criteria were as follows: (1) willingness to participate; (2) good dental health with functional dentitions; (3) no congenital orofacial abnormalities; (4) no prior history of neurological or psychiatric problems; (5) ability to follow study instructions; (6) normal or corrected-to-normal vision; and (7) no known facial impairment or orofacial pain that could interfere with facial expression or affective motor behavior. The exclusion criteria were predefined based on an earlier study by Kokich et al. [33] and additionally included the following: (1) an alexithymia score greater than 62 on the Toronto Alexithymia Scale (TAS) [34]; (2) decayed or missing teeth, sans 3rd molars; (3) orofacial defects or abnormalities; (4) prior history of organic or congenital diseases; (5) reported neuromotor or musculoskeletal impairments that would interfere with smile production (e.g., as described by [35]); and (6) reported use of medications with known motor side effects, e.g., abnormal involuntary movements or extrapyramidal symptoms. Sample characteristics are displayed in Table 1.

### 5.2. Research Protocol

The study protocol was approved by the Academic Committee of the Institute of Signal and Information Processing at Southwest University, which monitors the ethics of research involving human subjects. This study was conducted in accordance with the Declaration of Helsinki revised in 1989. Based on the protocol, data from three female participants were removed from the analyses (two persons are self-reported having the alexithymia scores of TAS greater than 62; another person reported with poor sleep at the night right before the experiment). All participants were instructed to read a brief description of the research and sign an informed consent form.

Each participant was informed about the three qualifier tests before the commencement of the study. The qualifier tests comprised of a learning session and a testing session. In the learning session, authors conducted face-to-face interviews with the participants to ensure that they felt comfortable with the procedure and had a full understanding of it.

The data from 23 participants (mean age ± SD = 21.6 ± 1.6), who met the inclusion and exclusion criteria described above, were used for further analysis.

### 5.3. Questionnaires and Interview

This study used two self-reporting questionnaires and one interview with an expert as qualifiers for the population. The two questionnaires are Toronto Alexithymia Scale (TAS) and Affect Questionnaire [36].

The TAS [34]—a measure of difficulty in differentiating, describing, and expressing emotions—was utilized to screen and exclude subjects who could not vividly express their emotional states (TAS score > 62). The TAS score of 62 is often considered as a valid, conservative cutoff point estimate of alexithymia in nonclinical populations [37]. Each item was scaled on a five-point Likert scale from 1 = strongly disagree to 5 = strongly agree; the TAS has a reliability of 0.82.

The Affect Questionnaire was used to test whether specific emotions are elicited effectively during arousal tasks. The questionnaire comprises the following 28 items of affect adjectives: happy, delighted, excited, astonished, aroused, tense, alarmed, angry, afraid, annoyed, distressed, frustrated, miserable, sad, gloomy, depressed, bored, droopy, tired, sleepy, calm, relaxed, satisfied, at ease, content, serene, glad, and pleased. Each subject was required to finish the questionnaire prior to, and following, each task. Each adjective item is then rated on a five-point scale (where 1 = very slightly or not at all, 2 = a little, 3 = moderately, 4 = quite a bit, and 5 = very much). The subjects who passed the two questionnaires were then interviewed by an expert trained in FACS.

The interview session was conducted by an expert trained in the facial action coding system (FACS) [38, 39]. The FACS is designed to exclude those subjects who do not show their facial expressions when feeling happiness.

Based on the results of the three tests, members of the population who scored at or above the required score participated in the study. To recap, this procedure was chiefly aimed at ensuring that subjects who participated in the study elicited smiles spontaneously when feeling happiness; subjects who displayed self-control in displaying emotional facial expression were excluded.

### 5.4. Stimulation Protocol

The emotion stimulation material is an important tool to induce experimental emotion. On the basis of the different channels, the existing emotion stimulation materials may be divided into visual stimulation materials, auditory stimulus materials, olfactory stimulation materials as well as the multimedia materials, and so on. Therefore, emotion stimulation research treated as an extraordinary issue has received more extensive attentions. Along with the deepening of emotion research, Gross and Levenson [40, 40] have found that film can induce the stronger emotional feelings and acquire more cognitive participation as an emotion elicitation way compared with other stimuli such as music, picture, and recall, etc. In this work, therefore, we choose short-length video clips for emotion elicitation.

The elicitation stimuli used in this work are some funny audiovisual video clips which are chosen by prior questionnaire surveys using the nine-point Likert scale. Meanwhile, validation results from 391 subjects also demonstrate that the materials could obviously elicit the human's emotions in some sense and help them achieve the desired movements [41]. During the selection of the film clips, the film plots which have aroused certain target emotion of the subjects are labeled as emotion-eliciting film plots. Once the experiment begins, the subject can only follow the experiment instructions which have been informed to the subject before. When the labeled film plots occur instantly (i.e., the emotion elicitation frames appear in time), it will trigger the capturing of HSI instrument by the start click operation of the experimenter. Then, in the process of emotion eliciting, the original data will be collected and transferred to a computer disk.

### 5.5. Experimental Setup

**Figure 1:**
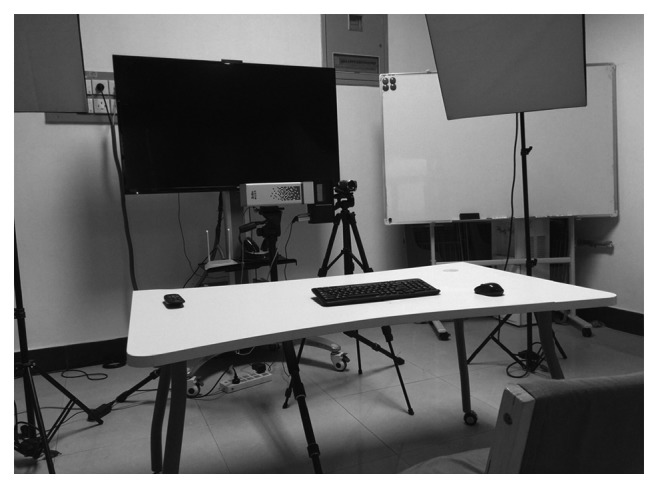
Experimental setup: hyperspectral data are recorded by an HSI camera while the subject smiles.

All subjects were seated comfortably in a brightly illuminated and acoustically and electrically restricted room. To capture affective data, an HSI camera (imaging spectrometer V10 E from SPECIM Inc, Finland; CCD from Lumenera Inc, Canada) was placed at about eye level 200 cm near the level of the subject's Frankfort horizontal plane. Additionally, after being synchronized for videotaping the visual data, a visual camcorder was aligned with the subject's midsagittal plane to provide a full frontal view of the face.  Figure 1 shows the experimental setup. The specified resolution of this camera is 1392 × 1040 pixels with a spectrum range of 400–1000 nm.

The subjects who passed the three qualifying tests were invited to participate in the experiment, which comprised of three sessions: Calm (for baseline data), Happiness Session 1, and Happiness Session 2. Two happiness sessions were employed to evaluate the reliability of the test.

**Figure 2:**
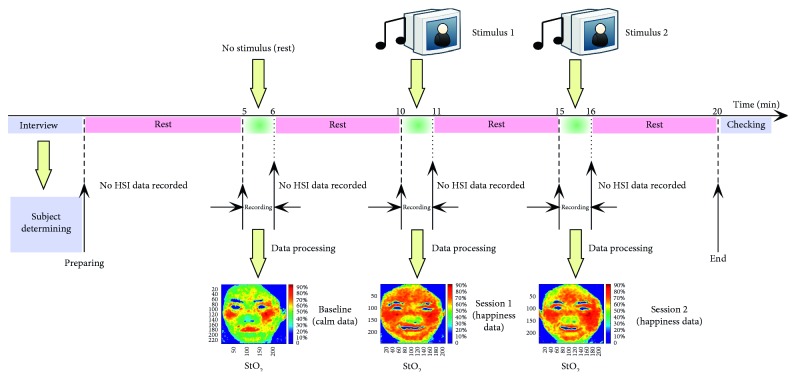
Procedure of experiment.

Before the test, each subject was required to rest for about 5 minutes. After the short rest, baseline data were collected while the subject remained “calm.” Then he/she would rest once again—this time for about four minutes. Subsequently, the test required the subject to be prompted to elicit spontaneous emotions of happiness by the stimulus material. Simultaneously, the instrument would collect and transfer the original data to a computer disk. The data transfer would last about four minutes depending on the amount of block data and the processing speed. Meanwhile, the subject was asked to rest quietly. After the data had been successfully saved, the subject would be instructed to embark on another happiness task, but this time with another stimulus material for the purposes of comparative analysis. Once again, the relevant data was stored on a computer disk. The whole experiment typically lasted for about 20 minutes per participant. The experimental procedure is illustrated in Figure 2.

## 6. Data Collection

### 6.1. Action Units

**Figure 3:**
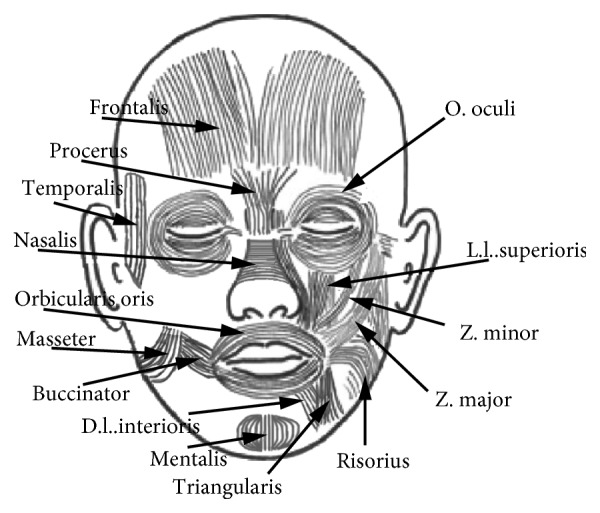
Distribution of the muscles implicated in the smiles.

In this study, the selected smiles were based mainly on certain distinct facial movements defined as action units (AUs). Most smiles essentially include the following AUs: (1) AU6 (“Cheek Raiser and Lid Compressor”), contraction of the orbicularis oculi; (2) AU10 (“Upper Lip Raiser”), contraction of the zygomaticus minor and levator labii superioris; (3) AU12 (“Lip Corner Puller”), contraction of the zygomaticus major; (4) AU20 (“Lip Stretcher”), contraction of the risorius; and (5) AU25 (“Lips Part”), relaxation of the lips and orbicularis oris in the mouth area. Besides the above AUs, smiles can more or less naturally trigger the movements of other muscles such as the depressor labii inferioris, mentalis, buccinator, masseter, nasalis, procerus, and temporalis because the muscles of the face function as a whole rather than individually. The distribution of muscles implicated in a smile is illustrated in Figure 3. In particular, AU6 (usually referred to as “Duchenne's marker”) has been highlighted as the primary unit to represent spontaneous smiles or genuine happiness [42]. Indeed, many studies have revealed that AU6 was observed when subjects genuinely experience more positive emotions such as happiness, and these same subjects also generated concomitant changes in neuromuscular movements [43].

### 6.2. Regions of Interest

In previous studies, researchers have selected different regions of interest (ROIs) to conduct extensive investigations in their respective study fields, using various advanced technologies. The analytical methods used in these investigations have achieved good results.

Pavlidis et al. [24] studied stress by measuring transient perspiratory responses in the perinasal area through thermal imaging. The results showed that different responses genuinely existed in human movements resulting from the manifestation of latent neurophysiological mechanisms. Chen et al. [30] utilized an HSI technique to detect stress in the forehead area. The accuracy for stress recognition from baseline using a binary classifier was 76.19% and 88.1% for the automatic and manual selections of the classifier threshold, respectively. Fischer et al. [44] analyzed the differences in the muscles (frontalis, nasalis, and orbicularis oris) between the lower face and upper face using focal transcranial magnetic brain stimulation. Kim and Provost [45] investigated the temporal characteristics of specific ROIs such as eyebrow, cheek, forehead, and mouth. This led the authors to conclude that combining different ROIs enhanced the overall accuracy of the findings.

### 6.3. HSI Data

**Figure 4:**
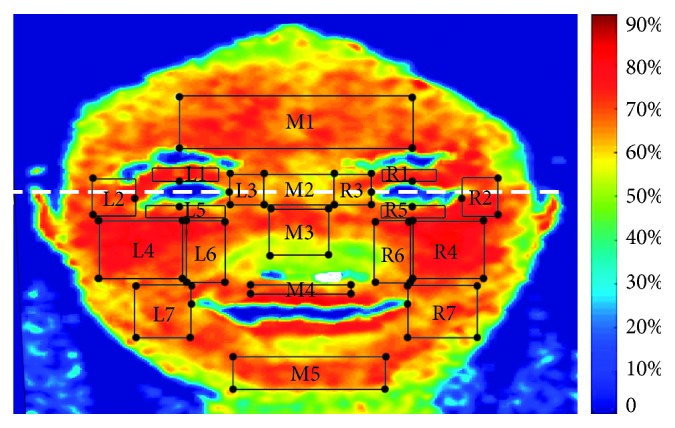
Regions of interest investigated.

Based on the AUs associated with the smile, the authors considered both the neuromuscular movements and the previous research findings of the characteristic correlations between ROI and psychophysiological reactions. A detailed correlation among ROIs, muscle groups, and AUs is shown in Table 2. The last column of the table lists studies which have explored the psychophysiological responses associated with each ROI. According to the literature, we located the positions of the corresponding ROIs manually for each participant. For example, for forehead M1, we identified its location manually (i.e., from the center of the left eyebrow to the center of the right eyebrow and from the top of the eyebrows to 1/2 the distance from the top of the eyebrows to the top of the head). Other regions of ROIs can be similarly determined according to the associated literature. To this end, 19 ROIs (*N*_ROI_=19) were investigated in this study; these are depicted in Figure 4. Each ROI was marked with a black rectangle. The ROIs contained unequal pixels owing to individual and regional differences.

This study aimed to quantify the neuromotor-controlled movements relating to happiness for further analysis. First, the reflective digit number of the human face using an HSI camera was obtained. According to the literature [57, 58], StO_2_ is considered as a psychophysiological signature to evaluate the effectiveness of differentiating the emotion states. Then, owing to differences in molar absorptivities in the subjects, the affective data (StO_2_) was computed using an optical absorption model [30–32, 59], which is cited as a scientific rationale. This study primarily used the Beer–Lambert law to calculate StO_2_ from HSI raw data. Here, StO_2_ is defined as the ratio of the amount of oxy-hemoglobin (HbO2) to the total amount of HbO2 and deoxy-hemoglobin (Hb) in body tissues.

The StO_2_ was calculated for 23 subjects for each ROI, which generates a subset of 23 data points for each ROI.  Table 3 shows the distributions of the StO_2_ variables.

## 7. Data Analysis

### 7.1. Descriptive Statistics

Based on a five-point Likert scale, the 23 participants completed their estimate of Affect Questionnaire by rating their arousal responses. In addition to the self-reporting scores of specific affect, we collected further data by calculating the composite scores to represent each of the four quadrants of the Circumplex Model [60] by summing the self-estimate ratings as follows: Quadrant 1: sadness (distressed, miserable, gloomy, bored, tired, sad, depressed, droopy, and sleepy); Quadrant 2: anger (alarmed, afraid, astonished, tense, angry, annoyed, and frustrated); Quadrant 3: relaxation (calm, satisfied, content, relaxed, at ease, and serene); and Quadrant 4: joy (happy, excited, aroused, glad, delighted, and pleased). The composite groups were mainly utilized to evaluate whether the targeted specific affect was aroused during each task session.  Table 4 shows the means and standard deviations of the composite groups and specific affects. Analysis of variance (ANOVA) revealed that the participants displayed significantly more intense feelings on the calm adjectives during the calm session and on the happy adjectives during both happiness sessions than the other three groups (*p* < 0.001). However, for the “joy” groups, no significant differences were found between the two happiness sessions (*p*=0.352 > 0.05). The authors therefore concluded that the stimuli could effectively elicit the subjects' happiness. For the specific affects, further analysis showed that there was no significant difference in scores between the males and females (*p*=0.577 > 0.05) with homogeneity of variance (*F*(1, 21) = 1.652, *p*=0.205 > 0.05). A paired *t*-test revealed that the arousal responses did not differ significantly within the two happiness sessions (*p*=0.327 > 0.05) and were highly positively correlated (*r*(45) = 0.494, *p*=0.01). The authors also concluded that the arousal responses were seemingly only influenced by the stimulus alone and not by other additional factors such as the interval and memory load.

### 7.2. Inferential Statistics

To examine whether the StO_2_ differed among the different emotion tasks (Calm, Happiness Session 1, and Happiness Session 2) for each ROI, multivariate analysis of variance (MANOVA) was performed using the emotion types (calm and happiness) as independent variables. First, the ln(·) transformation was used to comply with analysis of variance assumptions.

Furthermore, in order to compare the changes in the subjects' emotional responses within different sessions, a modified baseline correction was applied to compensate for the individual differences by transforming to comparable scales (changing rate) based on a calm state for a given subject. The ln(·) transformation was applied to comply with analysis of variance assumptions. As a result, two-way repeated measures ANOVA was conducted using ROIs as independent variables.

The results showed that the error variances of the dependent variables were equal across the groups, as evaluated by Levene's test. A Greenhouse–Geisser correction for nonsphericity was applied if Mauchly's test of sphericity was significant. For simplicity, the whole facial region was divided into three parts by sagittal plane: left region (LR); middle region (MR); and right region (RR). The LR comprised L1, L2, L3, L4, L5, L6, and L7; the MR included M1, M2, M3, M4, and M5; and the RR was made up of R1, R2, R3, R4, R5, R6, and R7. The results discuss these three parts: analysis for each ROI, within-region analysis, and between-region analysis.

## 8. Results and Discussion

First, MANOVA is used to analyze the main effect of the emotion factor for each ROI. Second, in the within-region analysis, the LR, MR, and RR are discussed separately to determine any significant correlations within them. Third, the between-region analysis explores the interrelationships between the LR, MR, and RR.

### 8.1. Analysis for Each ROI

For the ROIs, MANOVA was performed for three sessions (Calm, Happiness Session 1, and Happiness Session 2). The comparable distributions of their correlations and differences are shown in Tables 3 and 5. In Table 3, it can be seen that the average intensity of StO_2_ was a little greater in Session 1 or Session 2 than in the Calm state for each ROI. In addition, the proportion of StO_2_ increased from 2.74% to 9.03% in Session 1 and varied within the range of 2.81%–9.10% in Session 2. Thus, the increase in fluctuations observed was in a similar range for both happiness sessions. Furthermore, it was found that the average values in the two sessions were very close.

An intuitive explanation is that these results are indeed all about smile processing and may have an approximately equal emotional expression in terms of neuromotor mechanism.

However, this is only a subjective judgment; further analysis will be evaluated and discussed in the following section. Moreover, another aspect for consideration is that since the individuals each have their own distinct differences in personalities, they may vary in their affective reactions so that StO_2_ as a physiological signal differs according to a given emotional state. The experimental results in Table 3 show that the 19 different regions do not exhibit the same reactivity for a specific state. At the baseline, L7 has the minimal value of 37.24%, and R4 has the maximal score of 54.72%. While emotions are elicited, they would be correspondingly enhanced.

**Figure 5:**
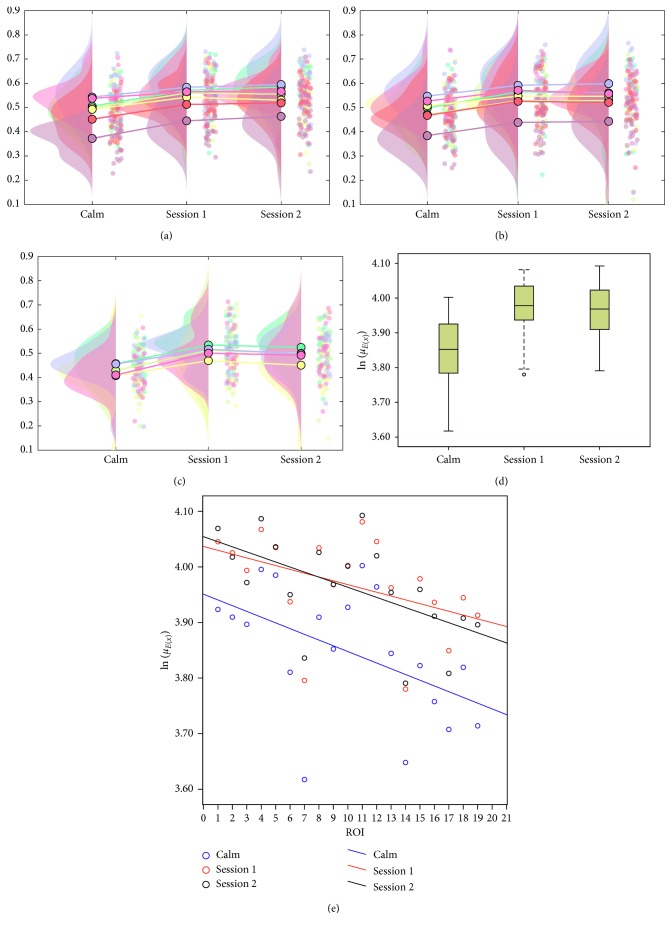
Distributions of StO_2_ for different ROIs during three sessions: (a) LR; (b) RR; (c) MR. (d) Box-plot diagram of the average StO_2_ during three sessions; (e) scatter plot of the average StO_2_ versus ROI for three sessions. The number of *X*-axis indicates the label of ROI. Note that we, respectively, denote by *E*(*x*) the average StO_2_ value of an entire ROI and by *μ*_*E*(*x*)_ the average StO_2_ intensity of all subjects under a certain ROI.

Figures 5(a)–5(c), respectively, describe the raincloud distributions of StO_2_ under three sessions (Calm, Session 1, and Session 2) for different ROIs such as LR, RR, and MR. These plots also specifically illustrate the individual differences of ROIs within each session group. They visually characterize the intuitive interrelations between individual and group distributions.  Figure 5(d) shows the distributions of average StO_2_ during the calm state and the two sessions. Using a Bonferroni-adjusted significance level (*α′* = 0.05/3 = 0.017), the analysis showed that the subjects displayed more happiness in the two task sessions than in the calm state (ANOVA, *p* < 0.017 for both happiness sessions). However, no significant difference in arousing happy feelings was found between the two task sessions (ANOVA, *p* > 0.017). This finding reflects not only the changes in the different ROIs for the same person for a given emotional state but also reveals the oscillation of StO_2_ during different states.  Figure 5(e) shows a scatter plot of StO_2_ versus ROI distributions. The results clearly show that the happiness sessions could not be distinguished from each other, but that both of these sessions could be distinguished from the calm state.

Subsequently, the authors tested whether the subjects had equivalent mean responses across the three sessions. For each ROI, there is a family of *n*=3 tests. Hence, the significance level *α* = 0.05 is Bonferroni adjusted to *α′* = 0.05/(3 ∗ 19) = 0.00088. The significant differences are shown in Table 3. It could be concluded that compared to the calm groups, the physiological indicators revealed significant happiness differences for most ROIs in both happiness sessions using a paired *t*-test (*p* < 0.00088), while there are no significant differences between Session 1 and Session 2 for the ROIs (*p* > 0.00088).

Additionally, another evaluation index (effect size, Cohen's *d*) was used.  Table 5 shows the correlations and divergences for the groups. The correlation coefficients between the two happiness sessions were greater than those between other sessions. Such intercorrelations indicate that if people smile happily, the range of increase in StO_2_ is proportional in each ROI.

After considering all of the above indicators, it was concluded that StO_2_ increased significantly in different periods for every ROI, with fewer overlapping regions between happiness and calm states and a similar increasing gradient for two individual changing stages from calm to happiness. Moreover, no significant variation in physiological signals was observed between the two happiness sessions with larger superposed areas. This suggests that arousal does not influence neuromuscular signals. Furthermore, for a specific emotional state, distinct differences in the physiological movements measured by StO_2_ were observed in all ROIs. These significant differences will be discussed in the following sections.

### 8.2. Within-Region Analysis

For the within-region analysis, the LR, MR, and RR were analyzed separately. In order to compare the subjects' happiness responses with calm responses on an equal footing, it was necessary to apply a modified baseline correction for every subject to calibrate the individual differences. The ln(·) transformation was used to ensure analysis of variance assumptions.

For each segmented region, LR, MR, and RR, and the whole region abbreviated as AR, two-way repeated measures ANOVA was used for the task type; these results are shown in Table 6. The results show that the interrelation between ROI and session was insignificant. Furthermore, the effects between the sessions are also insignificant, but ROI is found to be highly significant in all the experiments.

**Figure 6:**
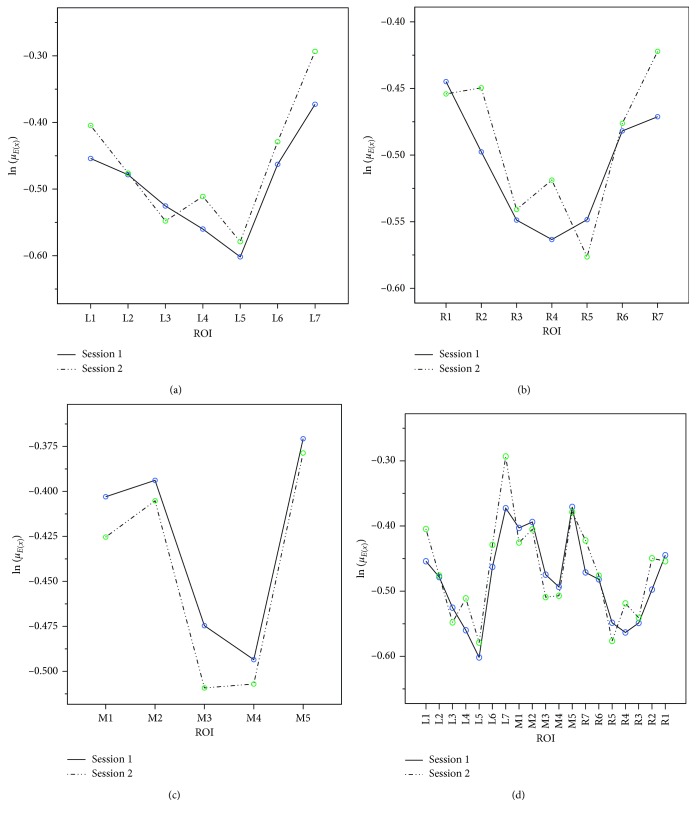
Session and ROI interaction plots for (a) LR, (b) RR, (c) MR, and (d) all regions. We used the ln(·) transformation to comply with analysis of variance assumptions.

First, for the LR, a post hoc analysis with a paired *t*-test for each session was performed and the results are displayed in Table 7. It was found that for both Sessions 1 and 2, L5 differed significantly from L1, L2, L6, and L7, and the difference was also significant between L4 and L7. The mean differences between the other tests had no statistical significance. Additionally, L3 was significantly different from the regions L1, L6, and L7, and a difference was also found between L1 and L4. The differences in other comparisons were not significant. These interrelationships are illustrated in Figure 6(a).

By observing the distribution patterns of StO_2_ for different sessions, no significant interaction effect (*p* > 0.05) was noticeable between ROI and session.

Second, the same analysis was performed for the RR, and the results are shown in Tables 6 and 7. In Session 1, R1 and R6 were both found to be significantly greater than the other regions R3, R4, and R5. The mean differences in the other tests were not statistically significant. However, in Session 2, R5 was significantly different from the other regions R1, R2, R6, and R7. The differences in the other comparisons were not significant.  Figure 6(b) illustrates these interrelationships. Once again, no interaction effects were observed between ROI and session. While minor differences between the two sessions in the ROIs were apparent (in R4, for example), none of these were significant for any of the ROIs (*p* > 0.05).

In terms of the symmetry characteristics of the LR and RR, the variation trends of the ROIs were identical which complied with an intuitive judgment. The intensity levels of the average StO_2_ of the corresponding ROI between the LR and RR—such as the StO_2_ of L4 in the LR versus the StO_2_ of R4 in the RR—were markedly different. One explanation of this is that the subjects' neuromuscular responses did not involve the same mechanism of action because of the emotion mode. For example, smile intensities were stronger in the left regions than in the right regions when the participants elicited genuine happy smiles. These interesting findings of partial asymmetry (lateralization of affective processing) have also been addressed in many studies [61–64]. They concluded the hemiface differences in visual exploration patterns when displaying genuine emotions. Lindell [61] reviews research examining asymmetries in the expression of facial emotion in humans, representing the right hemisphere's dominance for emotion processing. More specifically, it is the right hemisphere that innervates the lower left hemiface, resulting in more intense expressions. Najt et al. [62] reevaluated empirical evidence with respect to three competing yet partly conflicting models (the Right Hemisphere Hypothesis, the Valence-Specific Hypothesis, and the Approach/Withdrawal model). Results from their investigations showed that they did not fully support the models, demonstrating a left hemisphere advantage for the perception of happy expressions and a right hemisphere advantage only for a subset of negative emotions including anger, fear, and sadness (rather suggesting a “negative valence model”). Prete et al. [63, 64] concluded that the right hemisphere was more responsive to emotional faces than the left hemisphere. Additionally, the authors presented that there was no correspondence between behavioral and electrophysiological results concerning asymmetries for emotion processing, and that the Valence-Specific Hypothesis and the Right Hemisphere Hypothesis were not mutually exclusive. Therefore, it is very complicated to evaluate the hemifacial asymmetries in expressivity.

An analysis of the MR produced similar results: the difference between the two sessions and the interaction effects between ROI and session were not significant (Table 6). With a paired *t*-test, comparing Session 1 and Session 2 and adjusting for Bonferroni's correction, the results were not significant. Subsequently, an ANOVA revealed significant differences between M5 and both M3 and M4 for Session 1 and Session 2, as shown in Table 7. The deviations of the mean differences in the other comparisons were not significant.  Figure 6(c) shows the interactions between session and ROI for the MR.

Not surprisingly, other than the partial asymmetries of the left/right hemiface, upper and lower parts of the face would also express the “partial asymmetries” for the perception of emotions [65–67]. Ross et al. [66, 67] claimed overwhelmingly independent motor control of the upper and lower face in the studies. In addition, they found evidence that the right and left face may also exhibit independent motor control, thus supporting the concept that spontaneous facial expressions are organized predominantly across the horizontal facial axis and secondarily across the vertical axis. Unlike the cognitive control of facial expressions for social purposes in the lower face, the upper face may often display/leak a person's true feeling state by producing a brief facial emotion. Meletti et al. [68] presented, by ERPs study, the response patterns of EEG with faces encoding happiness and fear in the eye region compared to those encoding emotions in the whole faces or in the mouth region. Zeng et al. [69] revealed that blocking the facial feedback of lower face significantly boosted the recognition accuracy of subtle and intense microexpressions under all duration conditions, highlighting the important role of applying the upper face in judging the subtle movements of microexpressions. As known from the above observations in the experiment, most of the upper ROIs demonstrate the significant signatures than the lower ROIs, which are almost consistent with the previous findings.

### 8.3. Between-Region Analysis

Having performed contrastive analysis for the three individual regions, we then needed to investigate the interactions among them. Following the above analysis, in order to effectively determine their deeper connections, we divided the regions into two groups according to the degree of significance, i.e., a high-StO_2_ correlation group (HCG) and a low-StO_2_ correlation group (LCG). Thus, L3, L4, and L5 of the LR and R3, R4, and R5 of the RR were grouped into the LCG, while the other subdivisions of the LR (L1, L2, L6, and L7) and the RR (R1, R2, R6, and R7) were included in the HCG. MR was not divided into groups. Owing to the specialty of the MR in the midsagittal plane and the symmetry of the LR and RR, we studied only the interrelations between the LR vs. the MR and the RR vs. the MR.  Figure 6(d) shows the distribution of the average StO_2_ for all of the ROIs.

Firstly, we performed a comparison analysis between the LR and MR. In the contrastive analysis between the LCG and the MR, two-way repeated measures ANOVA revealed that the interaction effect was not significant (*p*=0.891 > 0.05) and the sessions had no effect on the variations in StO_2_ (*p*=0.929 > 0.05). However, the main effect of the ROI was significantly different (*p*=0.0005 < 0.05). After adjusting for Bonferroni's correction, the results of our post hoc analysis showed that L5 was significantly lower in StO_2_ than M1, M2, and M5 in Session 1 (*p*=0.0002 < 0.05 for M1, *p*=0.04 < 0.05 for M2, and *p*=0.015 < 0.05 for M5), with M1 significantly higher than both L3 and L4 (*p*=0.045 < 0.05 for L3 and *p*=0.010 < 0.05 for L4). In Session 2, only M1 showed a significant difference with L3 or L5 (*p*=0.018 < 0.05 for L3 and *p*=0.045 < 0.05 for L5). Next, we used ANOVA between the HCG and the MR, which demonstrated that the interaction effect was also insignificant (*p*=0.929 > 0.05), with session insignificant (*p*=0.883 > 0.05) and ROI significant (*p*=0.0004 < 0.05). However, the differences in ROI between the two sessions were no longer significant after adjusting for Bonferroni's correction. With no adjustment in the least significant difference (LSD), we found that both M1 and M5 showed more significant intensities in StO_2_ than L2 in Session 1 (*p*=0.027 < 0.05 for M1 and *p*=0.034 < 0.05 for M5), with L7 more significant than M1 (*p*=0.033 < 0.05), M3 (*p*=0.015 < 0.05), and M4 (*p*=0.015 < 0.05) in Session 2.

We then performed a comparison analysis between the RR and MR. Firstly, we conducted an analysis on the LCG and the MR. ANOVA revealed that the interaction effect was insignificant (*p*=0.851 > 0.05), with session also insignificant (*p*=0.885 > 0.05). However, the main effect of ROI was significantly different (*p*=0.0004 < 0.05). In Session 1 after Bonferroni correction, M1 was found to have a significantly greater StO_2_ than either R3 or R4 (*p*=0.001 < 0.05 for R3 and *p*=0.036 < 0.05 for R4). Also, StO_2_ was significantly greater in M5 than in R3 (*p*=0.006 < 0.05), R4 (*p*=0.014 < 0.05), and R5 (*p*=0.034 < 0.05). Furthermore, in Session 2, after Bonferroni correction, R3 was significantly lower in StO_2_ than the ROIs (M1: *p*=0.010 < 0.05, M2: *p*=0.016 < 0.05, and M5: *p*=0.015 < 0.05). Moreover, the StO_2_ of R5 was also significantly lower than M1, M2, and M5 (*p*=0.025 < 0.05 for M1, *p*=0.032 < 0.05 for M2, and *p*=0.013 < 0.05 for M5). Subsequently, the test study between the HCG and the MR verified an insignificant interaction effect (*p*=0.996 > 0.05) and session effect (*p*=0.994 > 0.05) and a significant ROI effect (*p*=0.025 < 0.05). Post hoc analysis shows that the differences in both Session 1 and Session 2 were insignificant after adjusting for Bonferroni's correction. We nevertheless applied the LSD test to compare each of the two groups. In Session 1, M1 and M5 both showed more significant happiness than R6 in terms of changing rate (*p*=0.026 < 0.05 for M1 and *p*=0.003 < 0.05 for M5), with M5 more significant than R2 (*p*=0.049 < 0.05). The differences in ROIs for Session 2 were all found to be insignificant.

### 8.4. Effects Size for Each ROI

**Figure 7:**
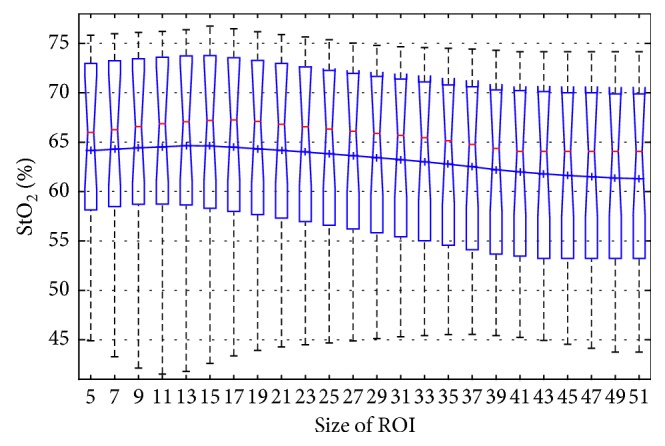
Distribution of StO_2_ over the size of ROI (N by N) for happiness state on the region of L4. The blue mark “+” represents the mean StO_2_ at the N by N scale. And the blue line denotes the connection line of the mean value in each N by N scale.

The size of the selected ROI determined the number of pixels involved in computing the average StO_2_ and subsequently further analysis. Generally speaking, the smaller the ROI, the fewer the number of pixels; therefore, higher variations in emotional signals would be generated. By increasing the size of the ROI appropriately, the variable effect was reduced and variations were uniformly diminished.  Figure 7 shows the distribution of StO_2_ for a random region L4. It illustrates that neither smaller nor larger ROIs could attain the maximum value of the average StO_2_. StO_2_ reaches an optimal average score for 13px-by-13px region. The average values obtained from the regions whose sizes were smaller than *N* = 13px almost reached the optimal value. This shows that the smaller regions had already included the most effective information. Only by significantly expanding the ranges could we acquire more extensive data; yet by continuing to enhance the ranges, more noises are introduced into the dataset. Moreover, the average range of values for the whole region only varied slightly—from 61.31% to 64.65%. Smaller ROIs mean higher oscillations which leads to an unstable distribution, while larger ROIs result in unnecessary noises becoming amplified in true signals. Both of the above scenarios are likely to lead to poor StO_2_ average values. Accurate results are achieved by using suitably sized ROIs. Regularities similar to those of L4 were observed with the other ROIs, and the observations were confirmed by validating the distribution of StO_2_.

## 9. Summary of Findings and Conclusions

Happiness as a psychological activity has attracted significant interest as a key area of research because it is associated with physical well-being. HSI technology, as a contact-free detection technique, is used to distinguish and quantify the amount of blood chromophores (Hb and HbO_2_). Subsequently, StO_2_, as a neurophysiological indicator, is considered to represent the affective response signals of neuromuscular activities.

In summary, this research shows that when people elicit genuine smiles, which indicate happiness, all of the facial regions are involved in displaying this emotion. However, there are significant variations in the degree of facial regions that show changes. The largest changes are seen in the upper eyelid, angulus oris, and mandible, which are controlled by the orbicularis oculi, risorius, and mentalis muscles, respectively. The forehead and ophryon exhibit the second largest variations in emotional intensities, managed by the frontalis and procerus muscles, respectively. The regions of the eye corner around the temple, oculonasal groove, nose, and perinasal area exhibit the second smallest variations in happiness measurements. The variations in the regions of the eye corner around the ophryon, lower eyelid (both controlled by the orbicularis oculi muscle), and cheek (controlled by the zygomaticus muscle) are least distinct. Thus, it may be concluded that although all the facial regions are more or less engaged in happiness and serve to create different effects, certain muscles, such as the orbicularis oculi, exert distinct effects on specific regions: the upper eyelid, lower eyelid, eye corner around the temple, and eye corner around the ophryon.

It was found that the regions involved in the observed expressions, as defined by action units (AU), produced statistically significant excitement based on the neurophysiological measurements. They include the regions of orbicularis oculi of AU6, zygomaticus minor and levator labii superioris of AU10, zygomaticus major of AU12, risorius of AU20, and orbicularis oris of AU25. Considering the above findings, it may be concluded that all of the above mentioned muscles show an increased degree of involvement when people display happiness. However, not all of them exhibit maximum variations.

For each ROI, the average intensities of StO_2_ increased significantly in both happiness sessions compared to the intensities found in the calm state as shown in the ANOVA results in Table 3. This indicated that the two arousal tasks prompted a statistically significant emotional response in the participants compared to the calm state (ANOVA, *p* < 0.017 for both happiness sessions). Additionally, the happiness intensities in the two sessions were not significantly different (ANOVA, *p* > 0.017). The results show that the physiological activities as a neuromotor mechanism have approximately equal emotional expression, and this is not affected by the time interval. Other evaluation indexes such as effect size (Cohen's d) and correlation coefficient also support the above mentioned conclusions.

To facilitate subsequent analysis, the whole facial region was divided into three parts: LR, MR, and RR, according to the sagittal plane. Both within-region and between-region interaction effects were studied separately to determine significant correlations among them, using two-way repeated measures ANOVA. It was found that the interaction effects between ROI and task factors were not significant. Only the ROI was found to be significant in all the experiments.

For the within-region analysis, in the LR segment, it was concluded that L1, L2, L6, and L7 could be divided into a group with higher variations in the changing rate of StO_2_ and L3, L4, and L5 into another group with lower variations. In the RR segment, though the variable regularities were not the same as for LR, similar general trends emerged. It was concluded that R1, R2, R6, and R7 form one group while R3, R4, and R5 form another group. One explanation of this nonuniform distribution could be that the subjects' neuromuscular responses did not involve the same mechanism of action because of the emotion mode. For example, by virtue of the hemiface differences caused by lateralization of affective processing, more neuropsychological responses may have occurred on the left side of the face than on the right side.

Lastly, in the MR, it was concluded that M5 showed a significantly greater changing rate than either M3 or M4 in both happiness sessions. Thus, M5 is a group in itself, while M1, M2, M3, and M4 form a second group.

For the between-region analysis, after adjusting for Bonferroni's correction and a LSD test, considering correlation differences in the changing rate of StO_2_, and taking into account the conclusions drawn from the within-region analysis, the authors concluded that the groups should be organized as follows: (1) L1, L7, and M5; (2) M1 and M2; (3) L2, L6, M3, and M4; and (4) L3, L4, and L5, with a similar organization in the RR. It is proposed that these grouping strategies be investigated further and may serve as a basis for further research.

## 10. Recommendations for Further Research

Genuine happiness manifests distinct responses among individuals which are measurable using remote-sensing technology. The blood-supply system in the facial muscles shows significant changes when people smile, and is suggested to be an indicator of genuine happiness. The authors will continue to further study this topic with the aim of learning more about, and modeling, patterns of happiness. In this work, we do conduct the experimental investigation only focusing on eliciting the genuine happiness. The interaction effects of the single psychological emotion on physiological activities have not been investigated in this experiment. Also, the contribution of neuromuscular activity (i.e., making a facial expression without any real emotion) still needs to be investigated further. Therefore, the individual contribution of how psychological emotion and neuromuscular activity affect the interactive performance of physiological reactions will be explored in the future work.

## Figures and Tables

**Table 1:** Demographic characteristics.

Characteristics	Mean (SD)
Age (years)	21.6 (1.6)
Education (years)	15.0 (1.0)
Body mass index	22.1 (3.0)
Alexithymia	50.3 (8.0)

**Table 2:** Association between ROIs, muscle groups, AUs, and related studies.

Symbol^a^	Quantity	Principal muscle groups	Associated AU of smile	ROI-related studies
L1, R1	Upper eyelid	Orbicularis oculi	AU6	Matzke et al. [46], Forte et al. [47]
L2, R2	Eye corner around temple	Temporalis	AU6	Ekman [42], Knoll et al. [48]
L3, R3	Eye corner around ophryon	Orbicularis oculi	AU6	Rajoub and Zwiggelaar [49], Pavlidis et al. [50]
L4, R4	Cheek	Zygomaticus	AU6, AU10, AU12	Kim and Provost [45], Kohler et al. [51]
L5, R5	Lower eyelid	Orbicularis oculi	AU6	Knoll et al. [48], Kashima et al. [52]
L6, R6	Oculonasal groove	Levator labii superioris	AU10	Matzke et al. [46], Whitton et al. [53]
L7, R7	Angulus oris	Composites of buccinator, masseter or risorius	AU12, AU20, AU25	Fischer et al. [44]
M1	Forehead	Frontalis		Kim and Provost [45], Chen et al. [30]
M2	Ophryon	Procerus		Finzi and Rosenthal [54], Vitti and Basmajian [55]
M3	Nose	Nasalis		Fischer et al. [44]
M4	Perinasal	Orbicularis oris	AU25	Pavlidis et al. [24]
M5	Mandible	Mentalis	AU25	Harrison et al. [56]

^a^L = left; R = right; M = middle.

**Table 3:** Distributions of StO_2_.

Symbol^a^	Baseline	Session 1	Session 2
	Calm (%)	Happiness (%)	Happiness (%)
L1	50.57 ± 7.96	57.13 ± 9.98^*∗*^	58.51 ± 8.59^*∗*^
L2	49.88 ± 7.57	56.00 ± 8.56^*∗*^	55.57 ± 9.10^*∗*^
L3	49.24 ± 7.89	54.25 ± 9.06^b^	53.08 ± 8.53^b^
L4	54.35 ± 8.66	58.40 ± 8.76^*∗*^	59.54 ± 9.68^*∗*^
L5	53.79 ± 6.81	56.53 ± 7.56^b^	56.60 ± 6.87^b^
L6	45.17 ± 7.60	51.28 ± 7.44^*∗*^	51.94 ± 8.44^*∗*^
L7	37.24 ± 7.15	44.51 ± 8.57^*∗*^	46.34 ± 9.01^*∗*^
R1	49.87 ± 9.21	56.49 ± 11.73^*∗*^	56.04 ± 10.59^*∗*^
R2	47.10 ± 7.90	52.88 ± 8.96^*∗*^	52.91 ± 11.93^*∗*^
R3	50.77 ± 7.90	54.73 ± 8.84^*∗*^	54.67 ± 8.20^*∗*^
R4	54.72 ± 8.91	59.21 ± 9.36^*∗*^	59.89 ± 10.40^*∗*^
R5	52.67 ± 7.23	57.15 ± 9.25^b^	55.70 ± 7.25^b^
R6	46.73 ± 7.85	52.58 ± 9.05^*∗*^	52.14 ± 9.45^*∗*^
R7	38.40 ± 7.85	43.82 ± 9.57^*∗*^	44.29 ± 10.83^*∗*^
M1	45.71 ± 7.19	53.44 ± 9.06^*∗*^	52.43 ± 8.73^*∗*^
M2	42.85 ± 9.80	51.23 ± 11.27^*∗*^	49.97 ± 10.59^*∗*^
M3	40.75 ± 10.32	46.96 ± 9.86^*∗*^	45.08 ± 10.93^b^
M4	45.57 ± 7.63	51.65 ± 7.40^*∗*^	49.79 ± 8.09^b^
M5	41.02 ± 7.99	50.05 ± 9.99^*∗*^	49.20 ± 10.33^*∗*^

Data shown as mean ± SD. ^a^L = left; R = right; M = middle. ^*∗*^The difference is significant at the *α*_A_ level by paired *t*-test, compared with calm group. *α*_A_ = 0.05/(3 ∗ 19) = 0.00088 (Bonferroni). ^b^The difference is significant at the 0.05 level by paired *t*-test, compared with calm group.

**Table 4:** Descriptive statistics.

	Composite groups				Specific affect
	Quadrant 1: sadness	Quadrant 2: anger	Quadrant 3: relaxation	Quadrant 4: joy	
Calm	8.10 ± 1.31	7.83 ± 1.43	21.65 ± 4.81^*∗*^	17.85 ± 5.16	3.61 ± 0.81
Session 1 (Happiness)	7.82 ± 1.40	8.16 ± 2.13	17.40 ± 5.11	21.25 ± 5.75^*∗*^	3.50 ± 0.75
Session 2 (Happiness)	7.95 ± 1.78	8.54 ± 2.11	18.13 ± 4.94	21.10 ± 5.16^*∗*^	3.34 ± 0.78

Data shown as mean ± SD. ^*∗*^*p* < 0.001.

**Table 5:** Correlation analysis and effect size.

		*r* value	Cohen's *d*
L1	C vs S1	0.752^*∗*^	0.727
	C vs S2	0.742^*∗*^	0.960
	S1 vs S2	0.887^*∗*^	0.148

L2	C vs S1	0.713^*∗*^	0.758
	C vs S2	0.723^*∗*^	0.679
	S1 vs S2	0.827^*∗*^	0.049

L3	C vs S1	0.445^a^	0.589
	C vs S2	0.595^a^	0.467
	S1 vs S2	0.855^*∗*^	0.132

L4	C vs S1	0.782^*∗*^	0.465
	C vs S2	0.793^*∗*^	0.565
	S1 vs S2	0.918^*∗*^	0.124

L5	C vs S1	0.605^a^	0.381
	C vs S2	0.574^a^	0.411
	S1 vs S2	0.810^*∗*^	0.010

L6	C vs S1	0.587^*∗*^	0.812
	C vs S2	0.606^*∗*^	0.843
	S1 vs S2	0.797^*∗*^	0.083

L7	C vs S1	0.655^a^	0.921
	C vs S2	0.505^a^	1.119
	S1 vs S2	0.832^*∗*^	0.209

R1	C vs S1	0.595^a^	0.628
	C vs S2	0.632^a^	0.622
	S1 vs S2	0.829^*∗*^	0.041

R2	C vs S1	0.572^a^	0.684
	C vs S2	0.610^a^	0.574
	S1 vs S2	0.738^*∗*^	0.003

R3	C vs S1	0.675^*∗*^	0.472
	C vs S2	0.748^*∗*^	0.484
	S1 vs S2	0.890^*∗*^	0.008

R4	C vs S1	0.718^*∗*^	0.491
	C vs S2	0.805^*∗*^	0.533
	S1 vs S2	0.852^*∗*^	0.068

R5	C vs S1	0.486^a^	0.540
	C vs S2	0.510^a^	0.418
	S1 vs S2	0.712^*∗*^	0.175

R6	C vs S1	0.738^*∗*^	0.690
	C vs S2	0.858^*∗*^	0.622
	S1 vs S2	0.822^*∗*^	0.047

R7	C vs S1	0.762^*∗*^	0.619
	C vs S2	0.736^*∗*^	0.623
	S1 vs S2	0.776^*∗*^	0.046

M1	C vs S1	0.516^a^	0.945
	C vs S2	0.560^a^	0.841
	S1 vs S2	0.897^*∗*^	0.113

M2	C vs S1	0.630^a^	0.793
	C vs S2	0.727^*∗*^	0.698
	S1 vs S2	0.842^*∗*^	0.115

M3	C vs S1	0.638^a^	0.615
	C vs S2	0.743^*∗*^	0.407
	S1 vs S2	0.764^*∗*^	0.180

M4	C vs S1	0.677^*∗*^	0.809
	C vs S2	0.466^a^	0.536
	S1 vs S2	0.555^*∗*^	0.240

M5	C vs S1	0.595^a^	0.998
	C vs S2	0.686^*∗*^	0.886
	S1 vs S2	0.797^*∗*^	0.083

C = calm; S1 = Session 1; S2 = Session 2. ^*∗*^The coefficient is statistically significant at the *α*_A_ level by correlation analysis. *α*_A_ = 0.05/(3 ∗ 19) = 0.00088 (Bonferroni). ^a^The coefficient is statistically significant at the 0.05 level.

**Table 6:** Two-way repeated measures ANOVA.

	LR^a^		RR^a^		MR^a^		AR^a^	
	*F* value	*p* value	*F* value	*p* value	*F* value	*p* value	*F* value	*p* value
ROI	19.669	≤0.001	7.685	≤0.001	4.246	0.006	21.291	≤0.001
Session	0.277	0.601	0.080	0.778	0.076	0.784	0.060	0.807
ROI*∗*session	0.529	0.783	0.444	0.845	0.049	0.995	0.368	0.985

^a^LR = left regions; RR = right regions; MR = middle regions; AR = all regions.

**Table 7:** Post hoc with paired *t*-test.

*t*-Value	Session 1 (Session 2)						
	L1	L2	L3	L4	L5	L6	L7
L1		0.758	1.501	2.816	5.637^*∗*^	0.187	1.563
L2	2.675		1.056	2.313	4.813^*∗*^	0.440	2.049
L3	3.531^*∗*^	1.600		0.689	1.736	1.318	2.701
L4	3.477^*∗*^	0.979	0.888		1.464	2.491	3.789^*∗*^
L5	6.796^*∗*^	3.443^*∗*^	0.640	2.418		3.709^*∗*^	4.603^*∗*^
L6	0.640	1.266	3.321^*∗*^	2.745	4.338^*∗*^		2.125
L7	1.888	3.244	4.040^*∗*^	4.014^*∗*^	4.801^*∗*^	2.903	

	R1	R2	R3	R4	R5	R6	R7
R1		1.014	2.992^a^	2.857^a^	2.546^a^	0.966	0.487
R2	0.082		1.083	2.010	1.359	0.364	0.564
R3	1.934^a^	1.440		0.431	0.008	2.755^a^	1.666
R4	1.463	1.843	0.550		0.475	3.118^a^	1.957
R5	3.170^a^	2.826^a^	0.819	1.866		2.251^a^	1.454
R6	0.504	0.513	1.919	1.438	2.866^a^		0.230
R7	0.493	0.424	2.072	1.787	2.354^a^	1.114	

	M1	M2	M3	M4	M5		
M1		0.214	1.351	1.301	0.691		
M2	0.612		1.337	1.200	0.464		
M3	1.342	1.728		0.313	2.489^a^		
M4	1.140	1.390	0.036		2.184^a^		
M5	0.907	0.598	2.703^a^	2.506^a^			

Upper triangular matrix denotes Session 1 and lower triangular matrix denotes Session 2. ^*∗*^The difference is significant at the 0.05 level (Bonferroni). ^a^The difference is significant at the 0.05 level (no adjustments).

## Data Availability

The data used to support the findings of this study are available from the corresponding author upon request.
